# IoT and ML approach for ornamental fish behaviour analysis

**DOI:** 10.1038/s41598-023-48057-w

**Published:** 2023-12-05

**Authors:** K. Suresh Kumar Patro, Vinod Kumar Yadav, Vidya S. Bharti, Arun Sharma, Arpita Sharma, T. Senthilkumar

**Affiliations:** 1 Fisheries Economics, Extension & Statistics Division (FEESD), ICAR-Central Institute of Fisheries Education, Mumbai, 400061 India; 2 Aquatic Environment & Health Management Division (AEHMD), ICAR-Central Institute of Fisheries Education, Mumbai, 400061 India; 3 Department of Computer Science and Engineering, Amrita School of Computing, Amrita Vishwa Vidyapeetham, Coimbatore, 641112 India

**Keywords:** Environmental social sciences, Climate-change impacts

## Abstract

Ornamental fish keeping is the second most preferred hobby in the world and it provides a great opportunity for entrepreneurship development and income generation. Controlling the environment in ornamental fish farm is a considerable challenge because it is affected by a variety of parameters like water temperature, dissolved oxygen, pH, and disease occurrences. One particular interesting ornamental fish species is goldfish (*Carassius auratus*). Machine learning (ML) and deep learning technique have significant potential in analysing voluminous data collected from fish farm. Through this technique, the fish farmers can get insight on feeding behaviour, fish growth patterns, predict diseases/stress, and environmental factors affecting fish health. The aim of the study is to analyze the behavioural changes in goldfish due to alterations in environmental parameters (water temperature and dissolved oxygen). Decision tree, Naïve Bayes classifier, K-nearest neighbour (KNN), and linear discriminant analysis (LDA) were used to analyse the behavioural change data. To compare the performance between all four classifiers, cross validation and confusion matrix used. The cross-validation error of LDA, Naïve Bayes classification, KNN and decision tree was 19.86, 28.08, 30.14 and 13.78 respectively. Decision tree was proved to be the most accurate and effective classifier. Different temperature and DO range were taken to predict fish behaviour. Some findings are, the behaviour of fish was rest between temperature 37.85 °C and 40.535 °C, erratic when temperature was greater than or equal to 40.535 °C, gasping when temperature was between 37.85 and 40.535 °C and when DO concentration was less than 6.58 mg/L. Blood parameter analysis has been done to validate the change in external behaviours with change in physiological parameters.

## Introduction

One of the most popular hobbies for reducing stress is keeping ornamental fish^1^. Around 1800 species of ornamental fish are available on the market, over 1000 of which are freshwater species. Over 120 countries are participating in the ornamental fish trade, and 90% of freshwater fishes are farmed, with the remaining 10% being harvested from the wild. Ornamental fish ranked as the 3110th most traded good in the world in 2020, with $330 M in global commerce. Ornamental fish exports fell by 2.1% between 2019 and 2020, from $337 million to $330 million. 0.002% of global trade is made up of ornamental fish trade (Observatory of Economic Complexity). In 2020 the top exporters of Ornamental Fish were Japan ($43.1 M), Indonesia ($38.5 M), Singapore ($33.8 M), Netherlands ($21.9 M), and Thailand ($21.9 M). In 2020 the top importers of Ornamental Fish were the United States ($63.7 M), China ($30.1 M), Germany ($23.5 M), United Kingdom ($22.2 M), and France ($19.4 M). One of the most cultivated ornamental species in India is the goldfish (*Carassius auratus* Linn.)^2^. Goldfish are currently mostly raised in open or flow-through systems. The intense culture of this species might be a viable solution to meet the species’ rising demand^3^.

Typically, goldfish live in warm, hypoxic, still waters with lush flora and muddy bottoms^4^. The critical temperature, or CTMax (Critical Temperature Maximum) or CTMin (Critical Temperature Minimum), is the point at which an animal loses its capacity to move from dangers that could eventually cause its demise^5^. There are many studies related to forecasting of water quality parameters with the help of machine learning models. Zhang et al.^6^ used kPCA-RNN method to predict water quality parameters. Barzegar et al.^7^ used CNN-LSTM model to predict pH and DO. But there are very few works related to classification of the behavioural change with respect to temperature and DO. Vanderzwalmen et al.^8^ worked to monitor water quality and fish health (physiology and behaviour) through a commercial supply chain for ornamental fish. They measured fish health indicators included mortality, wounds, waterborne cortisol, and behavioural alterations. Colchen et al.^9^ studied on the effects of temperature on the inter-individual relationships and group structure in a fish and they found that the inter-individual distances increased with increasing temperature, particularly the nearest neighbour distance. By integrating active and passive models of sensory and social information processing Harpaz et al.^10^ predicted individual fish behaviour in a group. Here, water temperature and dissolved oxygen were used to predict the gold fish behaviour under different conditions. Fish are usually classified as warm water with optimum growth above 20 °C or cold water with optimum growth below 20 °C^11^. Dissolved oxygen concentration is the most critical water quality parameter in fish culture. According to Ref.^12^, warm water fish growth depends on the concentration of DO in water. Most aquatic animals are healthiest and grow fastest when dissolved oxygen concentrations are near air saturation. The haemoglobin (cyanoglobin in crustaceans) becomes completely saturated when dissolved oxygen concentration reaches near saturation^11^. DO depletion occurs mainly twice a day, i.e., in the early morning and the evening and during this time, fishes coming on surface of the water for oxygen. Hypoxia in fishes occurs due to heavy depletion of DO.

For this study, IoT (Internet of Things) technology was used to collect, gather and summarize the data (water temperature and DO) in real time. The network of physical “things” known as the Internet of Things (IoT) enables these objects to gather, exchange, and distribute data. These objects include devices, instruments, systems, and other items that are embedded with electronics, software, sensors, and network connectivity^13^. Data collection, real-time image acquisition, wireless transfer, intelligent processing, and alert information release are all functions of this system. Neetha et al.^14^ worked on IoT and machine learning technology to build a smart aquaculture system and stated that IoT and machine learning are quickly evolving, with applications in various industries. Kiruthika et al.^15^ proposed an embedded system for autonomous fish farming control. The suggested remote monitoring of a fish farming system was using the Internet of Things (IoT) for real-time monitoring and control. Tawfeeq et al.^16^ focused on monitoring aquaculture farms so that farmers can discover problems early and take appropriate measures to maintain optimum conditions for the fish by employing factors such as temperature, pH, and turbidity using an IoT-based smart system as the working body.

A subfield of artificial intelligence (AI) and computer science is Machine Learning (ML), combines data and algorithms to simulate how people learn, gradually increasing the accuracy of the results^17^. In order to forecast without being programmed, ML algorithms develop a model based on sample data (training data). Machine learning research allows machines to acquire new knowledge, new skills, and reorganize existing knowledge^18^. Important tasks in machine learning are classification, regression, clustering, dimensionality reduction (DR), transcription, machine translation, anomaly detection, synthesis and sampling, and estimation of probability density and probability mass function. To do these tasks, the machine learning models are decision tree, Naive Bayes, Support vector machine, Artificial neural network, K nearest neighbour (KNN), Deep learning, and Ensemble learning^19^. Rashid et al*.*^20^ worked on prediction of the water quality of bio-floc by using parameters like temperature, pH, dissolved oxygen, ammonia, and total dissolved solids using different ML predictive models like Artificial Neural Network (ANN), Group Method of Data Handling (GMDH), Support Vector Machine (SVM), Least Squares Support Vector Regression (LSSVR), and Long-Short Term Memory (LSTM),found that only LSTM showed better accuracy (82%) than their model. Zhou et al.^21^ worked to detect the abnormal behaviour of the fish using hand-made 1000 verification behaviour videos and RNN model. The average accuracy of the model was 89.89%. The ML prediction of changes in external behaviour with respect to changes in physiological/water quality parameters can be supported/validated by a simple blood parameter analysis. Blood offers significant profile to study environmental impact on fish. Some of the blood parameters are haemoglobin (Hb), red blood cell (RBC), hematocrit (HTC), mean corpuscular volume (MCV), mean corpuscular haemoglobin (MCH), mean corpuscular haemoglobin concentration (MCHC), and white blood cell (WBC). These parameters were used to study the physiological changes in goldfish due to increase in temperature (hyperthermic condition).

There are many works related to overall change in behavioural pattern due to changes in water quality parameters, but very few works on the behavioural change classification, that to be in real-time water temperature and dissolved oxygen data. To find the behavioural changes in real-time water temperature and dissolved oxygen, this study has been done. The study will give users an understanding of how IoT devices operate in ornamental fish tanks. The study will also help in understanding the behaviour changes of fish due to change in water temperature and dissolved oxygen concentration and how machine learning techniques and algorithms work and how the user will get benefit from them. Also, behaviour changes were validated with blood parameter analysis.

## Materials and methods

### Experiment design

**Figure 1:**
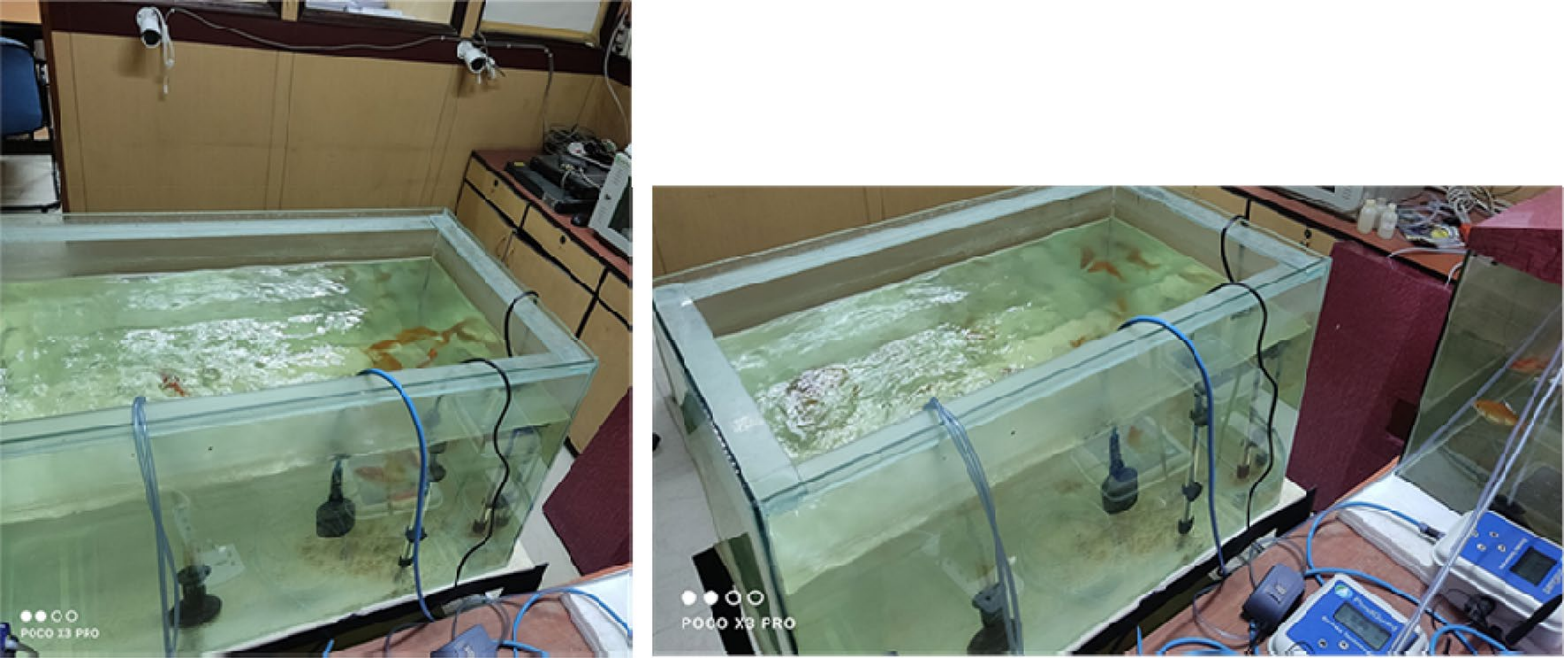
Experimental setup.

The dimension of experimental fish tank was 2.5 × 1.5 × 1.5 cubic feet. Goldfish were procured from the pet shop, and they acclimatized for a week with a 50% water exchange daily basis. The IoT sensors (water temperature and dissolved oxygen) were implemented in both the treatments to monitor and regulate the water quality parameters. For the behaviour record and object detection purpose, two Bullet Network Camera of model Hikvision DS-2CD206WFWD-I 6 MP IR were procured for behaviour change study purposes. The camera is featured with night vision (Fig. 1). The ranges of water temperature, pH and dissolved oxygen were 28–29 °C, 8.0–8.8 pH and 3.0–7.0 mg/L respectively (except for the time when temperature and DO were altered to check the behavioural changes). According to Ford and Beitinger^22^, the pH and DO are not influencing the temperature tolerance of fish. Goldfish were given feed with a frequency of once per day and left starved 24 h before the experiment.

The fish were continuously monitored by digital bullet camera with night vision to capture their behavioural changes. Ford and Beitinger^22^ have taken the exposure time interval of 8 h, 12 h, and 24 h. Fry et al.^23^ have taken the exposure time of 14 h. In this study, the temperature exposure time was up to 8 h with an increase of 2 °C in each 8 h interval. The initial temperature was 28 °C, and the critical upper limit temperature was 42 °C, where fish died. Interestingly the lethal temperature recorded by Fry et al.^23^ was 41 °C, and Ford and Beitinger^22^ recorded the upper limit temperature as 43.6 °C, so we can say that according to the existing literature and the study experiment, the maximum tolerable temperature level of goldfish is around 41–43 °C.

At a time, ten goldfish selected for the test and behaviour were recorded in camera with the real-time temperature and dissolved oxygen data which were recorded and distributed through IoT sensors. The behaviours were recorded along with real-time temperature and DO to perform some machine learning classification tools. Four classification tools were used to analyse the data, i.e., decision tree classifier, Naïve Bayes classifier, K nearest neighbour classifier, and linear discriminant analysis.

### Data collection and analysis

Real-time data of water quality parameters were used in this study. Internet of Things (IoT) technology used to collect the data real-time. The Internet of Things (IoT) basically describes the network of physical objects (things) that are embedded with sensors, software, and other technologies for the purpose of connecting and exchanging data with other devices and systems over the internet. In this study IoT sensors were used, which could able to measure DO and water temperature in real-time. Different machine learning classifiers were used to classify the behavioural change data as taking temperature and DO as independent variable and behavioural change data as dependent.

**Figure 2:**
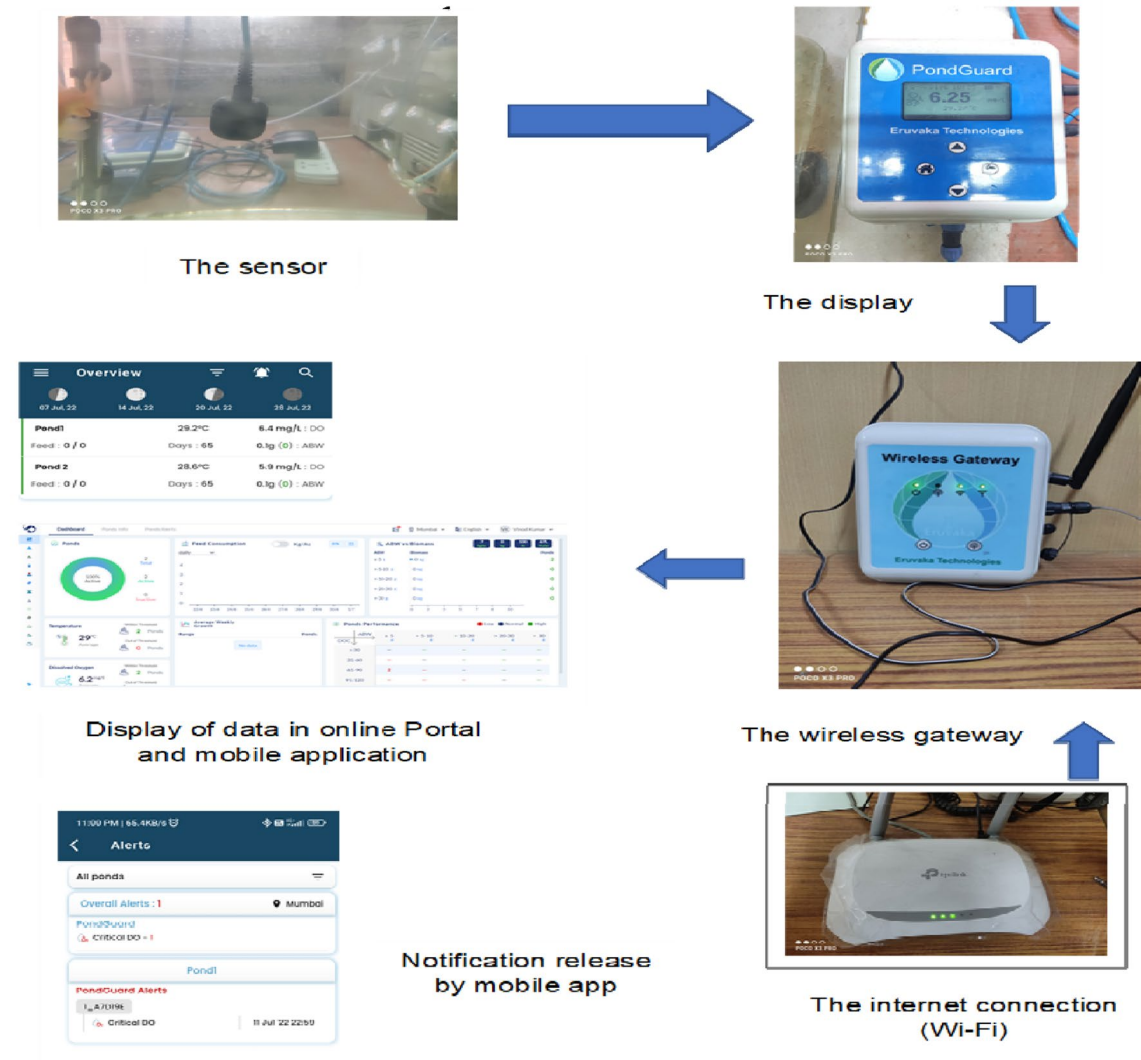
Flow diagram of deployment of IoT technology and flow of data.

Figure 2 represents the flow diagram of deployment of IoT technology. Here, the sensor will collect data from the environment, and the data will display in the display (PondGuard). There is always communication between the display and the wireless gateway, and the wireless gateway needs 24 h internet connection to get connected to the display. After getting the internet connection from Wi-Fi, the wireless gateway will transmit the data to the cloud, and from there, the data will come to the mobile app and the online portal for PC use.

The mobile app has the facility to create a water temperature and DO critical lower and upper limit; according to the environment, threshold limits are fixed; if the water temperature and dissolved oxygen concentration cross the threshold limits, then a notification with the date and time will come to the mobile app after that one can manage the situation easily without any manual intervention. The online site possesses a special feature to download all the recorded real-time data in excel format; one can download every single minute’s data from the beginning of installation in excel format; with this feature, one can see the trend and pattern of water temperature and dissolved oxygen in the farm.

### Decision tree

Decision trees, one of the most efficient data mining techniques, were first developed in the 1960s and are now widely employed across a variety of fields^24^, because they are simple to use, unambiguous, and robust even when a value is absent. It is possible to use target variables or independent variables that are both discrete and continuous. Common usages of decision tree models include variable selection, assessing the relative importance of variables, prediction, classification, etc. Nodes and branches make up the majority of a decision tree model, while splitting, stopping, and pruning are the key modelling operations^25^. Yeganeh-Bakhtiary et al.^26^ used M5p Decision Tree (DT) algorithm, a new and advanced model to predict wave characteristics. Yeganeh-Bakhtiary et al.^27^ developed decision tree (DT) models which were employed to statistically downscale the Beijing Normal University Earth System Model (BNU-ESM) global climate model output for prediction of Wind Characteristics under Future Climate Change Scenarios.

**Figure 3:**
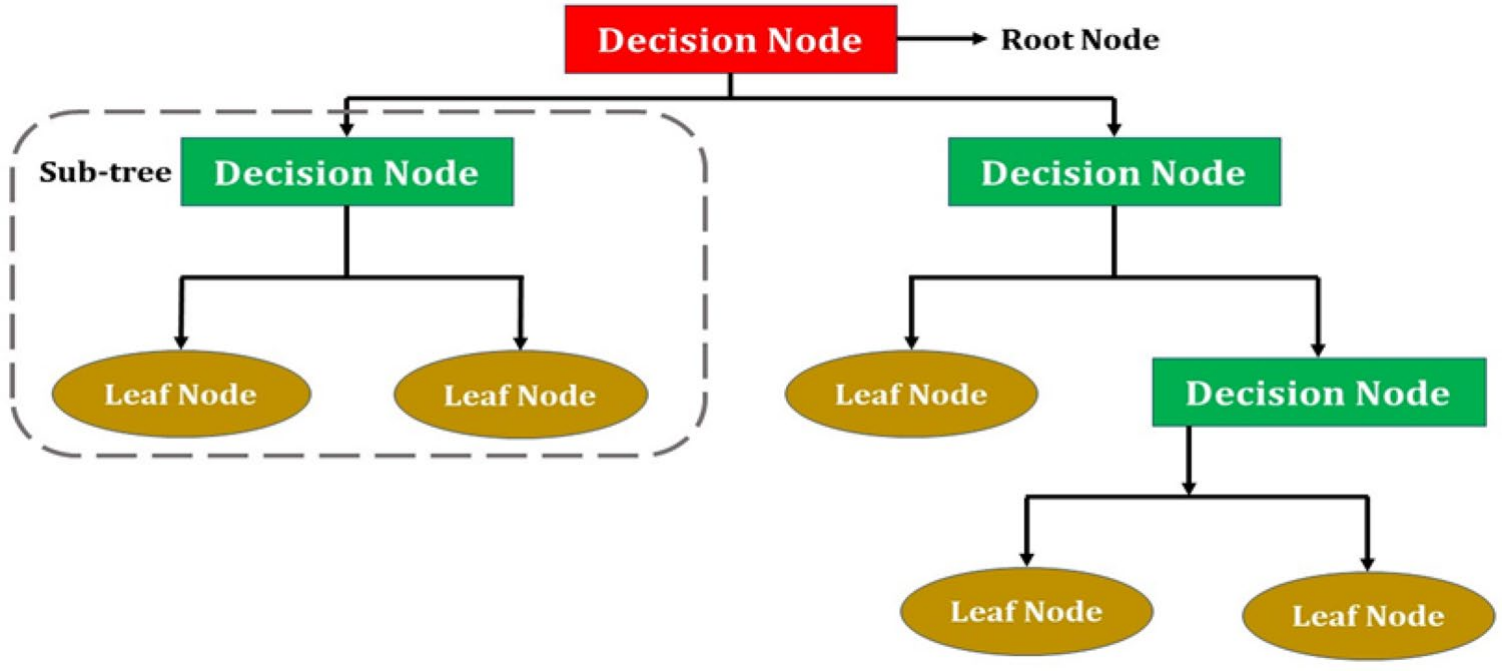
Basic structure of a decision tree.

It is a graphical depiction for obtaining all feasible answers to a decision or problem based on predetermined conditions. It is called as a decision tree because its structure is like a tree; it begins with the root node and grows on subsequent branches to form a structure resembling a tree. To construct a tree CART (Classification and Regression Tree) algorithm is used. A decision tree only poses a question and divides the tree into subtrees according to the response (Yes/No). Figure 3 represents the basic structure of a decision tree.

#### Working procedure of decision tree

##### Splitting

To divide parent nodes into purer child nodes of the target variable, only input variables relevant to the target variable are employed. It is possible to employ both continuous input variables that are collapsed into two or more categories and discrete input variables^25^. Entropy, Gini index, classification error, information gain, and gain ratio are some of the characteristics that are used to select between various potential input variables, these characteristics are related to the level of “purity” of the resultant child nodes^28^. Until predetermined homogeneity or stopping criteria are satisfied, this splitting procedure is carried out.

##### Stopping

When creating a decision tree, stopping rules must be used to keep the model from being too complex. The minimum number of records in a leaf, the minimum number of records in a node before splitting, and the depth (i.e., number of steps) of any leaf from the root node are common parameters used in halting rules^25^.

##### Pruning

Stopping rules do not always function correctly. A different approach to creating a decision tree model is to first construct a big tree, then prune it to the right size by deleting nodes that don’t provide much more information^29^.

Habib et al.^30^ worked on accurate prediction of wave overtopping at sea defences remains central to the protection of lives, livelihoods, and infrastructural assets in coastal zones by using different machine learning models and they explained the procedure of decision tree in simple six steps, i.e., (1) Data Preparation and Splitting, (2) Bootstrap Sampling, (3) Building Ensemble Decision Trees; (4) Training, Validation and Prediction on Training Set; (5) Hyperparameter Tuning; (6) Deployment to the test set.

#### Classification tree

According to James et al.^31^, classification trees and regression trees are extremely similar, except that classification trees are used to predict a qualitative response (a discrete category) as opposed to a numerical value (quantitative values). Both numerical and categorical input variables may be employed in the categorization process. The mean response of the training observations belonging to the same terminal node determines the anticipated response for observation in regression trees. For classification, the anticipation was made that each observation belongs to the class of training observations that occurs the most frequently in the region to which it belongs. Building a regression tree and a classification tree both include comparable steps. The classification tree is grown using the recursive binary strategy, just like the regression tree, according to James et al.^31^, although splits cannot be made using the residual sum of squares (RSS) in the classification tree setting. The classification error rate, which is just the percentage of training observations in that region that do not belong to the most prevalent class, is a superior alternative to the RSS approach. The classification error is given by,

1 $$E=1-max{\widehat{p}}_{mk},$$

where *p̂mk* represents the proportion of training observations in the region m that are from class k.

### Naïve Bayes classifier

A simplistic probability classifier, the Naive Bayes method determines a set of probabilities by counting the frequency and combinations of values in a given data set. Considering the value of the class variable, the algorithm applies Bayes’ theorem and assumes that all variables are independent. The method typically learns quickly in a variety of controlled classification problems, despite the fact that this conditional independence assumption is considered naive because it is rarely true in real-world applications^32^.

The mathematical method known as the Bayes’ theorem, which is named after the British mathematician Thomas Bayes, is used to calculate conditional probability.

2 $$P\left(A|B\right)=\frac{P\left(A\right) P(B|A)}{P(B)}.$$

Using the Bayes theorem and strong (naive) independence assumptions, a Bayes classifier is a straightforward probabilistic classifier. The phrase “independent feature model” might be a better way to describe the underlying probability model. Simply put, a naive Bayes classifier makes the assumption that the presence (or absence) of one character inside a class has no bearing on the presence (or absence) of any other feature.

The naive Bayes classifier’s advantage is that it only needs a small quantity of training data to estimate the parameters (variable means and variances) required for classification. Just the variances of the variables for each class must be calculated, not the entire covariance matrix, due to the assumption of independent variables. This model and a decision rule are combined by the naive Bayes classifier. The corresponding classifier is the function classify defined as follows:

3 $$Classify\left({f}_{1},\dots \dots ,{f}_{n}\right)=\genfrac{}{}{0pt}{}{argmax}{c} p(C=c)\prod_{i=1}^{n}p\left({F}_{i}={f}_{1}|C=c\right).$$

This implies that you should add the conditional probabilities of each feature given the class label for each possible class label. This indicates that all we need to do to build the classifier is to compute the individual conditional probabilities, p(Fi|Cj), for each label and each feature and then multiply them by the prior probability, p, for that label (Cj). The label the classifier returned is the one for which we received the greatest product.

### Linear discriminant analysis (LDA)

Linear discriminant analysis (LDA) is a generalisation of Fisher’s linear discriminant. It is also called normal discriminant analysis (NDA) or discriminant function analysis. It is possible to utilise the resulting combination as a linear classifier or, more frequently, to reduce the dimensionality before further classification. Regression analysis and ANOVA (Analysis of variance), which both aim to express one dependent variable as a linear mixture of other traits or measures, are closely connected to LDA^33, 34^. LDA and PCA and factor analysis are linked in that they both seek out linear combinations of variables that provide the most comprehensive explanation of the data^35^.

Fisher’s linear discriminant: It’s usual to use the words Fisher’s linear discriminant and LDA interchangeably, even though Fisher’s original work actually offers a considerably different discriminant that does not make some of the assumptions of LDA, such as normally distributed classes or equal class covariances. When groups are already known, discriminant analysis is employed (unlike in cluster analysis). Both a score on a group measure and a score on one or more quantitative predictor measures are required for each case. Discriminant function analysis is essentially classification, which is the process of grouping objects into types-specific groups, classes, or categories.

### K-nearest neighbour

**Figure 4:**
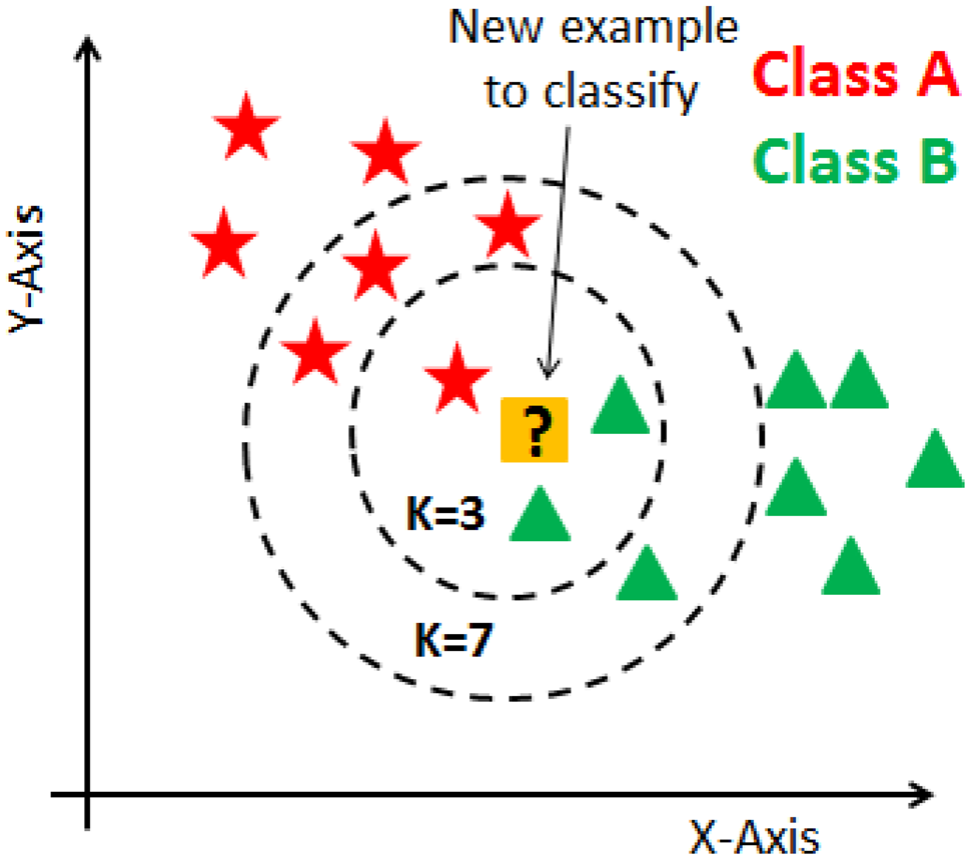
K-nearest neighbour classification example for K = 3 and K = 7.

Fix and Hodges introduced a non-parametric method for pattern classification that has since become known as the k-nearest neighbour rule^36^. The k-nearest neighbour algorithm is a non-parametric, supervised learning classifier. It employs proximity to classify or anticipate how a set of individual data points will be arranged. Although it can be applied to classification or regression issues, it is commonly employed as a classification algorithm because it relies on the idea that comparable points can be discovered close to one another. A class label is chosen for classification problems based on a majority vote, meaning that the label that is most commonly expressed around a particular data point is adopted. Despite the fact that this is official “plurality voting”, literature more frequently refers to “majority vote”. Similar to classification problems, regression issues use the concept,however, in this case, the average of the k nearest neighbours is used to forecast a classification^37^. Figure 4 represents a basic example of a K-NN model.

### Confusion matrix

**Figure 5:**
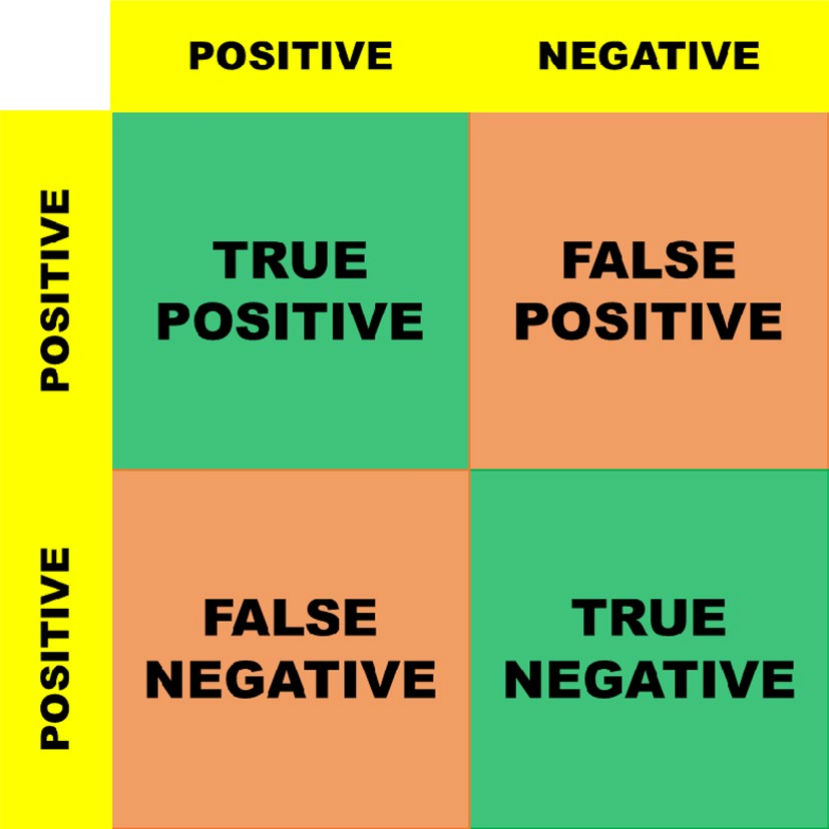
Confusion matrix.

A confusion matrix is a matrix that provides an overview of how well a machine learning model performs on a given set of test data. With the goal of predicting a categorical label for every input instance, classification models are frequently evaluated using this technique. Confusion matrices are helpful in problems involving multiclass and binary classification. The matrix can be used to compute a wide range of performance indicators. It is useful for statisticians and data scientists to understand these measurements^38^. A classifier’s predicted and actual values can be combined in four different ways (Fig. 5):

1. *True Positive (TP)* The number of times our actual positive values are equal to the predicted positive. You predicted a positive value, and it is correct.
2. *False Positive (FP)* The number of times our model wrongly predicts negative values as positives. You predicted a negative value, and it is actually positive.
3. *True Negative (TN)* The number of times our actual negative values are equal to predicted negative values. You predicted a negative value, and it is actually negative.
4. *False Negative (FN)* The number of times our model wrongly predicts negative values as positives. You predicted a negative value, and it is actually positive.

To find how accurate our model is, the following metrics were calculated:

1. *Accuracy* The accuracy is used to find the portion of correctly classified values. It tells us how often our classifier is right. It is the sum of all true values divided by total values.
  4 $$Accuracy=\frac{TP+TN}{TP+TN+FP+FN}.$$
2. *Precision* Precision is used to calculate the model’s ability to classify positive values correctly. It is the true positives divided by the total number of predicted positive values.
  5 $$Precision=\frac{TP}{TP+FP}.$$
3. *Recall* It is used to calculate the model’s ability to predict positive values. “How often does the model predict the correct positive values?”. It is the true positives divided by the total number of actual positive values.
  6 $$Precision=\frac{TP}{TP+FN}.$$
4. *F1-Score* It is the harmonic mean of Recall and Precision. It is useful when you need to take both Precision and Recall into account.
  7 $$F1 \, Score=\frac{2\times precision\times recall}{precision+recall}.$$

### Cross validation

Cross-validation is a technique used for resampling data to evaluate the generalization capacity of prediction models and avoid overfitting^39^. It is generally applied in situations where predicting outcomes is the major objective and one wishes to assess how well a predictive model would work in real-world scenarios. Like the bootstrap, cross-validation belongs to the family of Monte Carlo methods. In a prediction problem, a model is usually given a dataset of known data on which training is run, and a dataset of unknown data against which the model is tested. Cross-validation aims to identify issues such as overfitting or selection bias and provide information on how well the model generalizes to a different dataset by assessing the model’s predictive power over new data that was not included in its estimation.

In this study k-fold cross validation was used. The original sample is randomly divided into k equal-sized subsamples, sometimes known as “folds”, in k-fold cross-validation. One subsample (k − 1) is kept as validation data to evaluate the model, and the remaining k − 1 subsamples are utilized as training data. After that, the cross-validation procedure is carried out k times, using a single validation set of data from each of the k subsamples. One can then create a single estimation by averaging the k outcomes. K-fold cross-validation uses the following approach to evaluate a model.

*Step 1* Randomly divide a dataset into k groups, or “folds”, of roughly equal size.
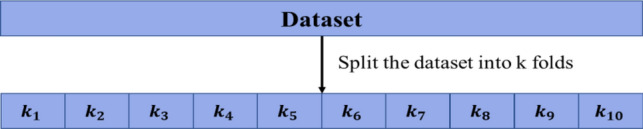


*Step 2* Choose one of the folds to be the holdout set. Fit the model on the remaining k-1 folds. Calculate the test MSE on the observations in the fold that was held out.
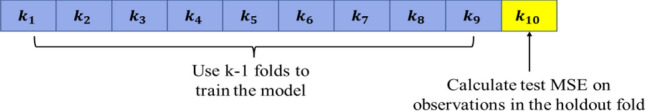


*Step 3* Repeat this process k times, using a different set each time as the holdout set.
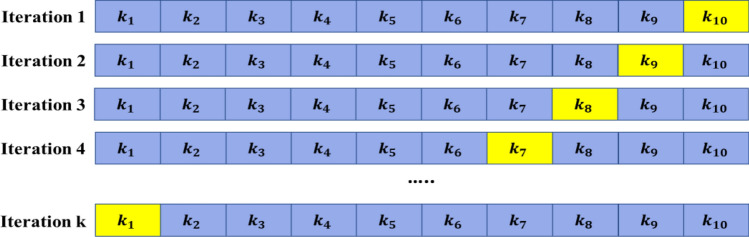


*Step 4* Calculate the overall test MSE to be the average of the k test MSE’s.

8 $$Test \,MSE=\frac{\sum MSE}{k}.$$

## Results

### Behaviour observation

To conduct this study 10 goldfish were used in each tank i.e., control and two treatments. Fish started to show some response after 36 °C; before that, fish were behaving normally. Categorization of three behaviours has been done while observing the recorded video, i.e., Gasping, rest at the bottom, and erratic swimming patterns.

**Figure 6:**
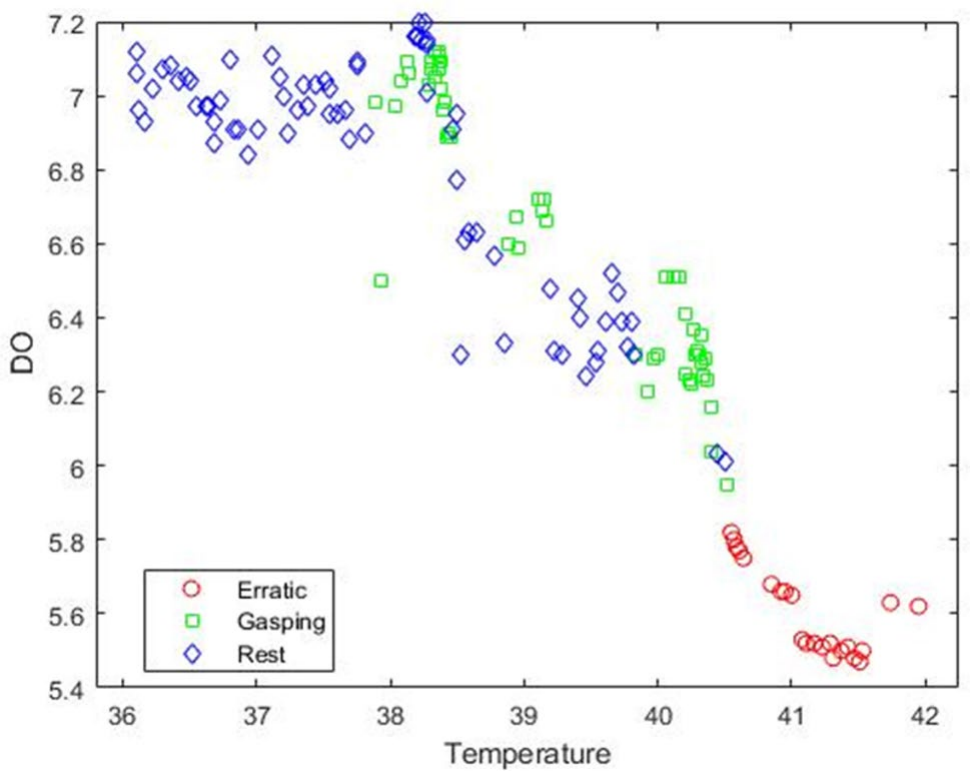
Scatter plot of behaviour dataset.

Table 1 represents the frequency of three behaviour observed during the temperature increase from 28 to 42 °C. Figure 6 represents the scatter plot for the behaviour data while raising the temperature. The three categories of behaviours were determined from previous studies (Table 1). Here, the red circle denotes erratic swimming pattern, the green square denotes gasping behaviour, and the blue rhombus denotes the rest at bottom behaviour. In X-axis, there is temperature and in Y-axis there is DO. The scatter plot shows the behaviours at a specific DO and temperature. It can be seen that most of the rest at bottom behaviour shows in between temperature 36 °C to 38 °C, the gasping behaviour is seen during the temperature 38 °C to 40 °C and the erratic swimming behaviour is seen during the temperature 40 °C to 42 °C.

**Table 1:** Description of the behaviours recorded.

Class	Class labels	Description	No. of instances of behavioural changes	Relation to welfare
1	Rest	Fish is in immobile condition	73	An indication of stress and anxiety is the evidence to freeze/rest^40, 41^
2	Gasping	Approximately all fish can be seen gasping at the water’s surface	51	Gasping is a sign of low DO levels^42^ and high temperature
3	Erratic	Fast swimming and changing of direction while not being attacked	22	Swimming erratically is an indication of increased stress, discomfort, or a pathogenic condition and could be interpreted as a sign of poor welfare^43–45^

### Classification of behaviour

**Figure 7:**
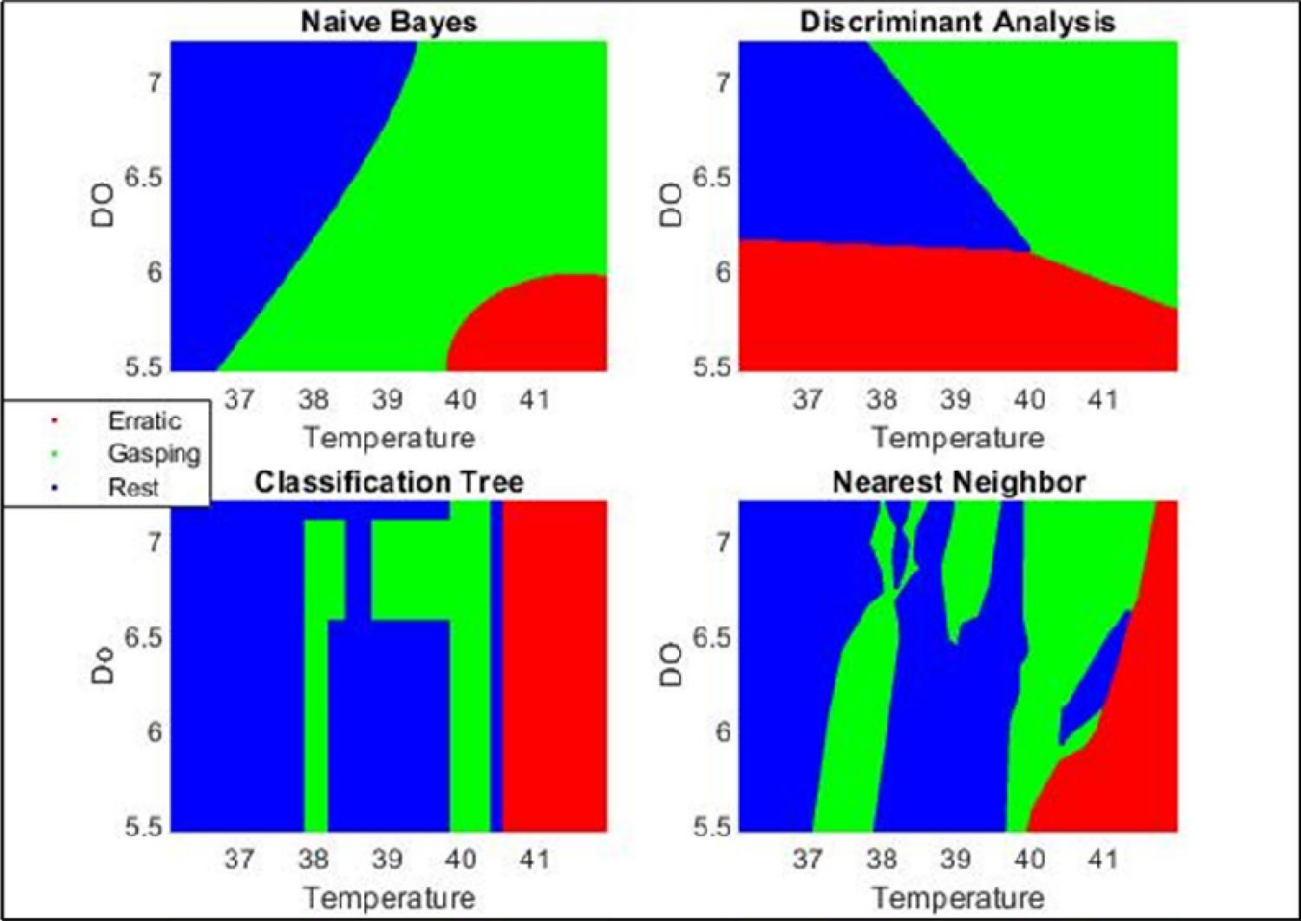
Classification comparison between Naïve Bayes, Linear discriminant analysis, decision tree and K nearest neighbour.

To classify the behaviour changes, the input or independent attributes were water temperature and dissolved oxygen, and the output or dependent attribute was behaviour change (rest, gasping and erratic swimming). 146 instances were recorded where most of the fish showed behavioural change and the behaviour change data was marked by real time water temperature and dissolved oxygen. To analyse the data four classifiers were used i.e., decision tree, Naïve Bayes classifier, linear discriminant analysis and K-nearest neighbour. Figure 7 represents the comparison between all four classifiers. To demonstrate the superiority of the decision tree methodology over Naïve Bayes classifier, linear discriminant analysis and K-nearest neighbour, the K (= 10) fold cross-validation method^24^ and confusion matrix were used. And the results of cross validation showed in Table 2. MATLAB 2022a software was used to carry out all machine learning analysis like scatter plot, K-fold cross validation, prediction, decision tree classification etc.

**Table 2:** The summary statistics of cross validation error of different classification methods after taking K (= 10) fold validation.

Method	Cross validation error/misclassification error (percentage)
Linear discriminant analysis	19.86
Naïve Bayes classification	28.08
K-nearest neighbour classification	30.14
Decision tree classification	13.78

The Table 2 represents the summary statistics of cross validation error of all four classification methods after taking k = tenfold validation. Linear Discriminant Analysis, Naïve Bayes classification, K-near neighbour classification, Decision Tree classification shows the cross-validation error as 19.86%, 28.08%, 30.14%, and 13.78% respectively. For collected dataset the decision tree is showing the best accuracy with cross validation error of 13.78%, and K-nearest neighbour is showing the lowest accuracy with cross validation error of 30.14%. According to Ozgonenel et al.^46^, decision tree classifier had the precision 0.96 compared to Naïve Bayes classifier (0.80) and Gaussian mixture model (0.92). Jadhav and Channe^47^, compared between decision tree, K-NN and Naïve Bayes classifier by using whether data and concluded that the accuracy of decision tree and KNN was more accurate (99%) compared to Naïve Bayes classifier which had the accuracy of 92.857%. Untoro et al.^48^ compared the Decision Tree, K-NN, Naive Bayes and SVM with MWMOTE on UCI Dataset and found that decision tree is an efficient process compared to K-NN, SVM and Naïve Bayes and concluded that for Decision Tree test data had an accuracy value of 94.32%, KNN of 92.67%, Support Vector Machine of 85.61%, and Naïve Bayes of 84.30%. Yadav et al.^49^ compared the fish abundance prediction accuracy between linear regression, neural networks and classification and regression tree (CART) models and found that NNs and CART models produced better prediction accuracy compared to LR model.

**Figure 8:**
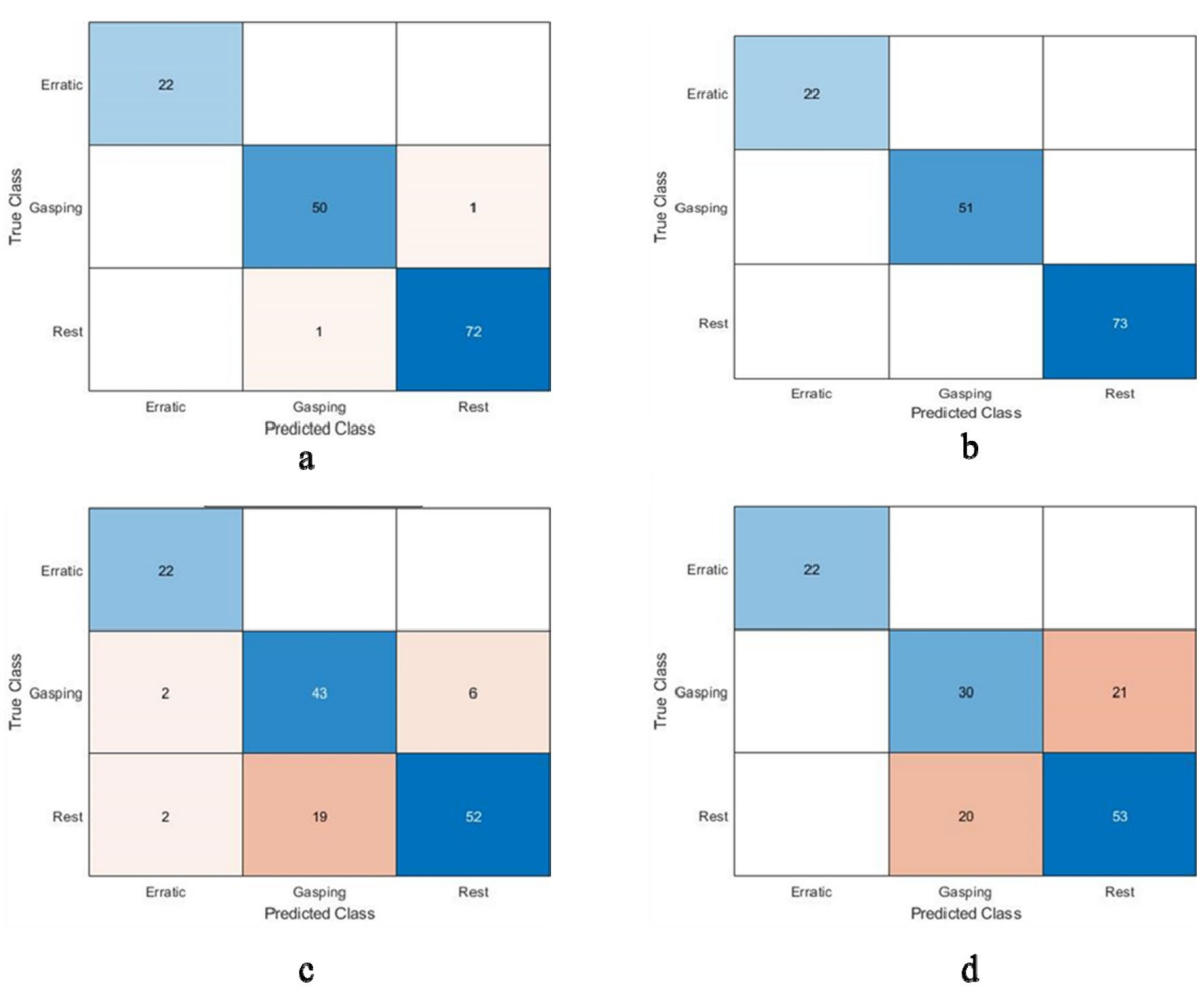
Confusion matrix for (**a**) decision tree, (**b**) K-nearest neighbour, (**c**) linear discriminant analysis, and (**d**) Naïve Bayes classifier.

Figure 8 represents the confusion matrices of all four classifiers i.e., decision tree, K-NN, linear discriminant analysis and Naïve Bayes classifier. As discussed earlier, there were three behaviours with some instances, there are 73 instances of rest behaviour, 51 instances of gasping behaviour and 22 instances of erratic behaviour. All confusion matrices have two classes namely true class and predicted class placed in Y-axis and X-axis respectively. Confusion matrix of decision tree is showing that the classifier predicted gasping behaviour as gasping behaviour in 50 instances and as rest behaviour in one instance, predicted rest behaviour as rest behaviour in 72 instances while as gasping behaviour in one instance and predicted all erratic behaviour as erratic behaviour. Confusion matrix of K-NN classifier is showing that the classifier predicted all three behaviours accurately. Confusion matrix of LDA is showing that the classifier predicted all erratic behaviour as erratic behaviour, out of 51 gasping instances the model predicted 43 instances as gasping; 2 instances as erratic and 6 instances as rest behaviour. Confusion matrix of Naïve Bayes classifier is showing that the model predicted all erratic behaviour as erratic, out of 51 gasping instances the model predicted 32 instances as gasping and 21 instances as rest, out of 73 rest instances the model predicted 53 instances as rest and 20 instances as gasping.

**Figure 9:**
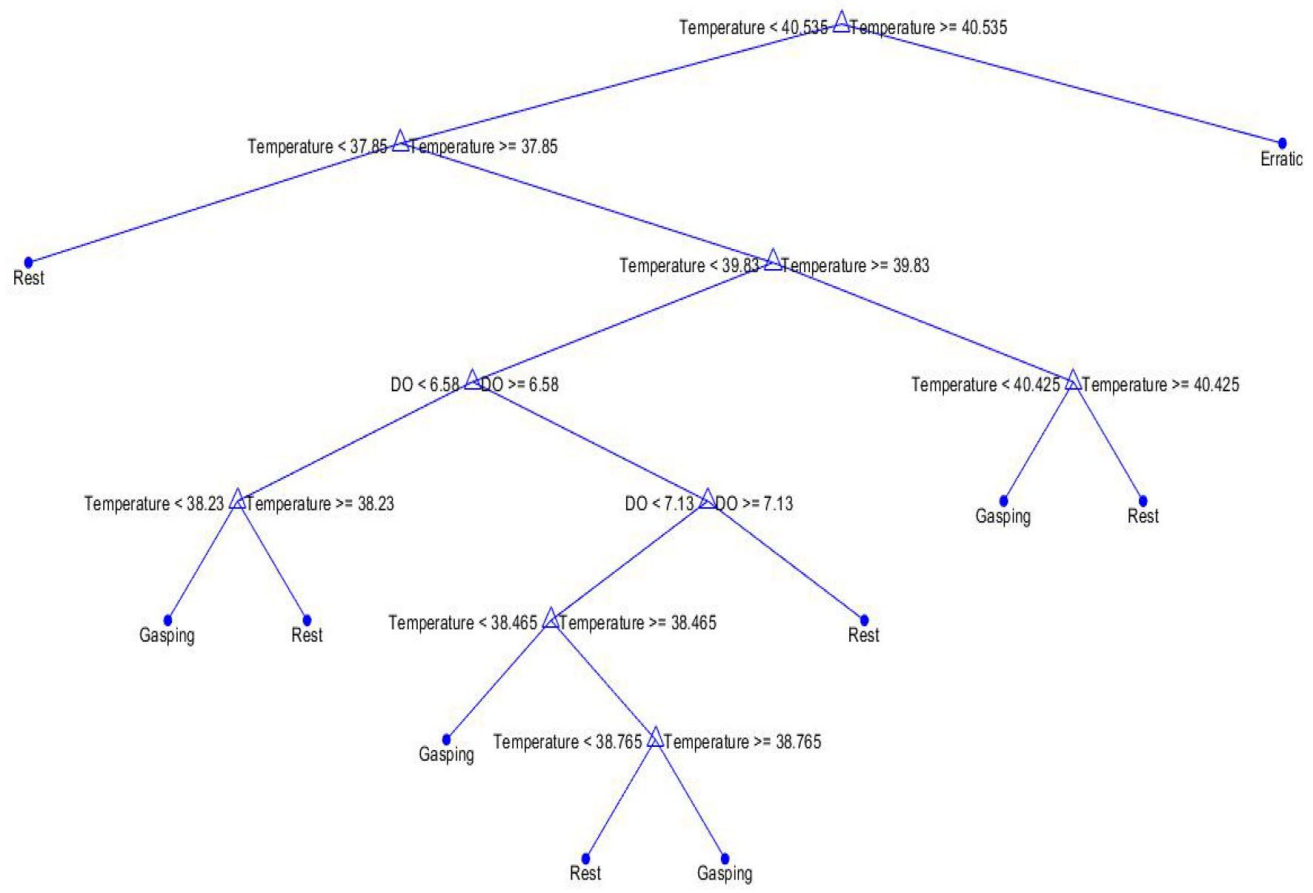
Decision tree classifier for behaviour data.

For validation, two approaches followed, i.e., K = tenfold validation method and confusion matrices. Findings from K = tenfold validation showed that the cross-validation error or misclassification error of decision tree was 13.78% which is quite less compared to other three, but findings from confusion matrix showed that K-NN had the best accuracy as K-NN predicted all three behaviour correctly and decision tree made two small errors, which can be neglected or we can say its accuracy was very nearer to K-NN. But according to K = tenfold validation method the cross-validation error of K-NN was 30.14% which is much more compared to DT with 13.78%. So, by observing these criteria the decision tree was selected as the classification model for this study. Figure 9 represents the decision tree for behavioural change data and Table 3 explains the summary of results of decision tree classifier.

**Table 3:** The summary of results of decision tree classifier.

Sl no.	Water quality parameters	Fish behaviour
1	Temperature < 40.535 °C and < 37.85 °C	Rest
2	Temperature ≥ 40.535 °C	Erratic
3	Temperature < 40.535, ≥ 37.85, < 39.83 and < 38.23, DO < 6.58	Rest
4	Temperature < 40.535 ≥ 37.85, < 39.83, DO ≥ 6.58, DO < 7.13 and temperature < 38.465	Gasping
5	Temperature < 40.535, ≥ 37.85, < 39.83, DO ≥ 6.58, < 7.13, temperature ≥ 38.465, < 38.756	Rest
6	Temperature ≥ 38.756	Gasping
7	Temperature < 40.535, ≥ 37.85, < 39.83, DO ≥ 6.58, ≥ 7.13	Rest
8	Temperature < 40.535, ≥ 37.85, ≥ 39.83, < 40.425	Rest
9	Temperature ≥ 40.425	Rest

**Figure 10:**
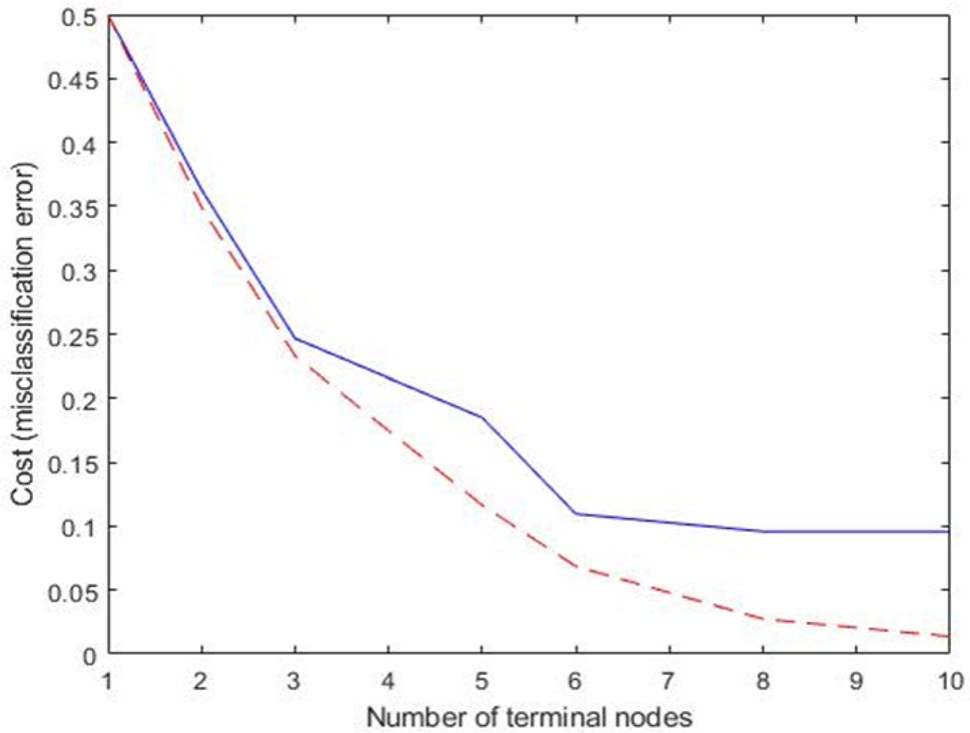
Error pruning level of decision tree.

In machine learning and search algorithms, pruning is a data compression approach that decreases the size of decision trees by deleting parts of the tree that are unnecessary and redundant for classifying occurrences. Figure 10 represents the error pruning level of decision tree used in this study. Pruning lowers the final classifier’s complexity, which increases predicted accuracy by reducing overfitting. Here the shortest distance between the train and test line is at terminal node number 6. For this reason, the proposed decision three has 6 terminal nodes. A study has been done on blood parameters of fish in both normal and test condition to validate the behavioural data, for showing that there was not only change in physical behaviours but also change in physiological behaviours.

### Blood parameter study of fish (hyperthermic and hypoxic condition)

Data pertaining to blood parameters for hyperthermic condition are presented in Table 3. Changes in the Hb content of blood in response to the environment might come about either by a change in the number of erythrocytes or by a change in the Hb concentration of the individual cells^50^. In this study there was increase in haemoglobin in test goldfish after water attaining temperature of 41 °C. In goldfish RBC diminished under acute and chronic thermal stress perhaps due to haemodilution as a consequence of osmoregulation^51^.

**Figure 11:**
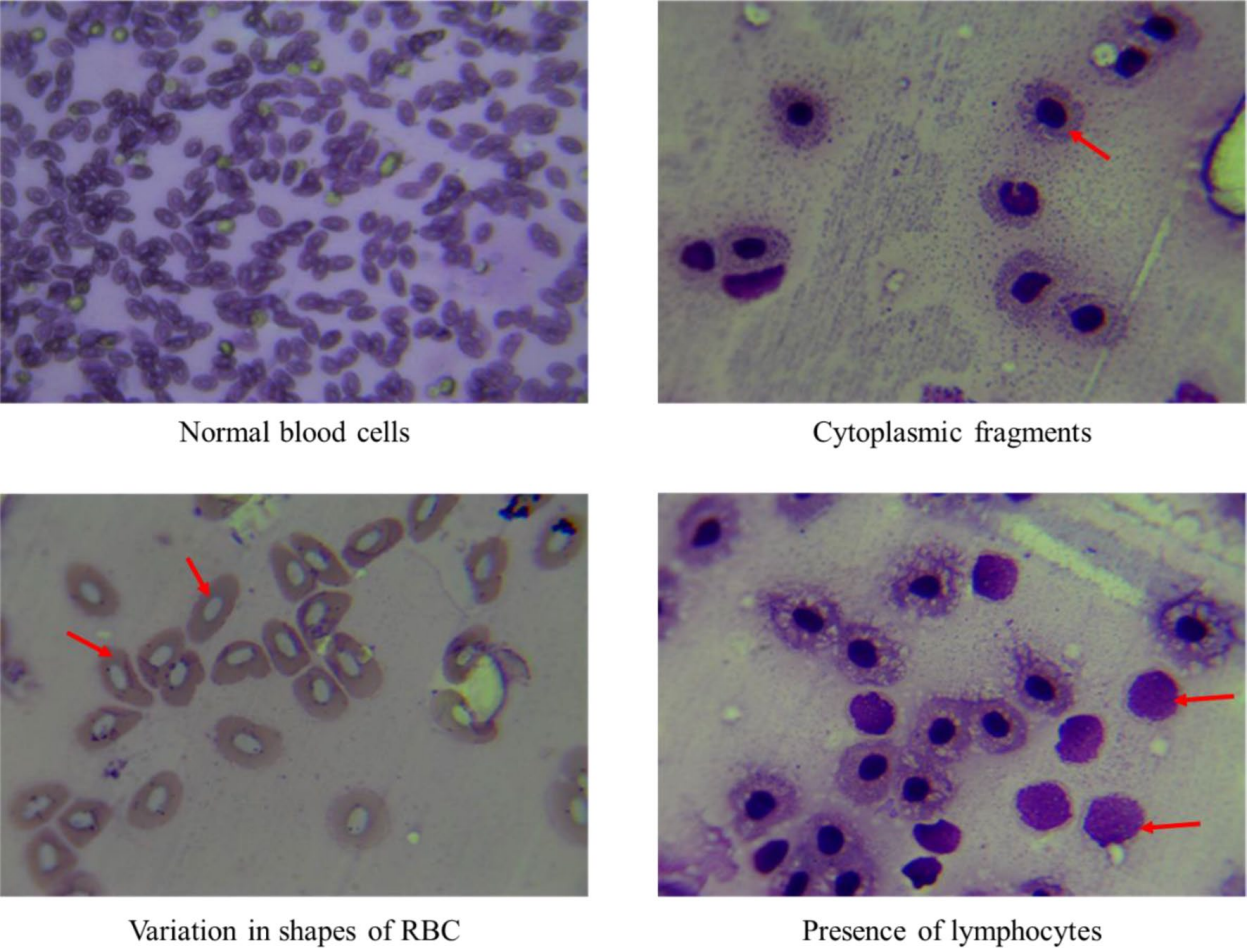
Pictures showing blood cells of goldfish from hyperthermic condition.

**Figure 12:**
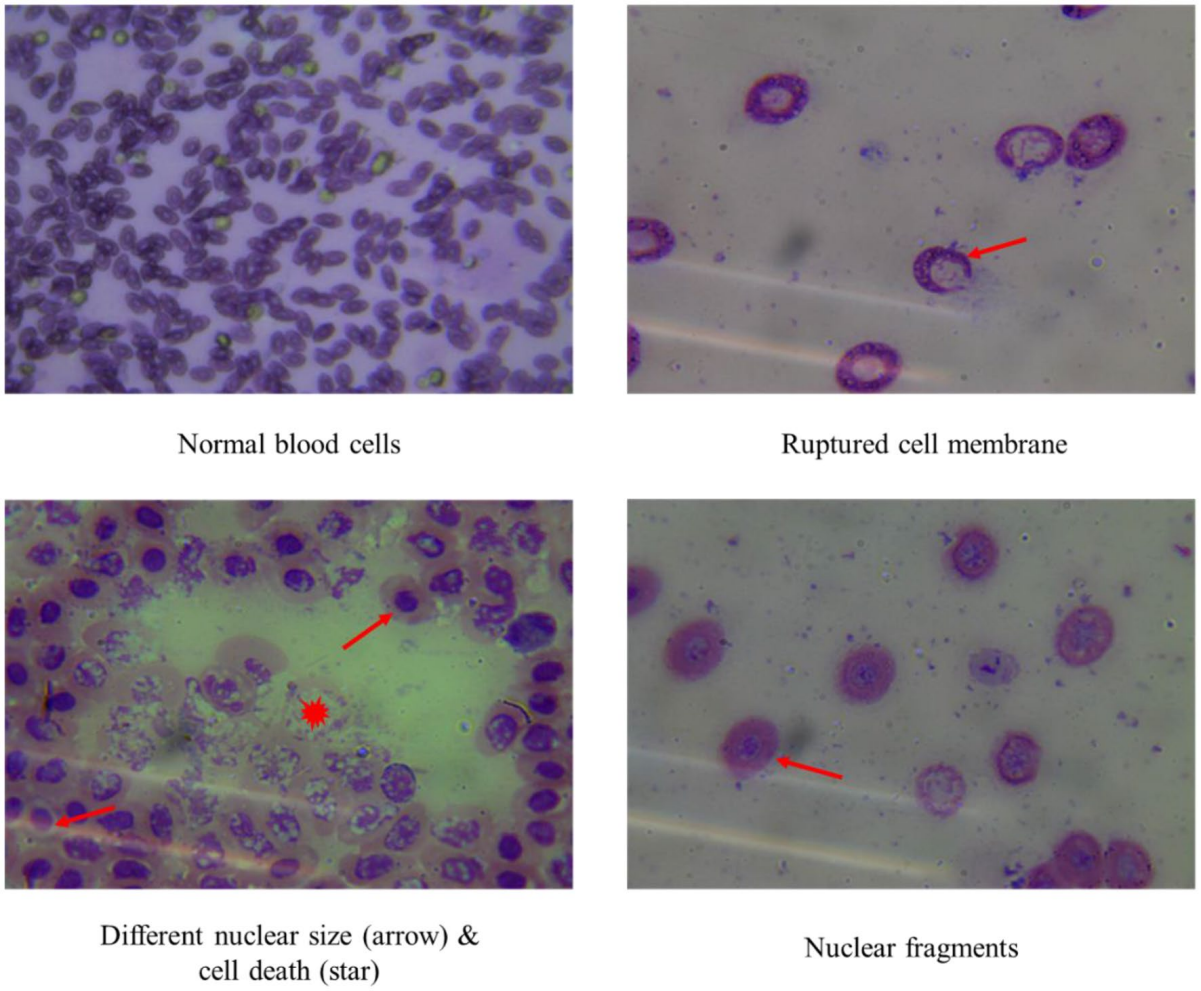
Pictures showing RBC of goldfish from hypoxic condition.

The dissolved oxygen concentration was reduced up to 0.5 mg/l and there were mainly two behaviours; gasping^52^ and sluggish movement^52^. When a fish gasps, it remains just below the surface, places its snout at the air–water interface, and inhales the film of water that is in direct contact with the air. In comparison, the oxygen content of this thin layer of water is high^52^. In sluggish movement behaviour most fish will greatly curtail their general activity^52^. Data pertaining to blood parameters for hypoxic condition are presented in Table 4. The hypoxic condition could induce anaemia by erythrocytes malformation and disruption, methaemoglobin formation, and others^53^. Here, there is an increase in haemoglobin concentration in hypoxic fish compared to normal fish. Figures 11 and 12 represents the alteration in blood cells when goldfish was in hyperthermic and hypoxic condition, respectively (Table 5).

**Table 4:** Blood parameters for hyperthermic condition.

Blood parameters	Control	Test
Haemoglobin	4.1 g/dl	4.8 g/dl
RBC count	0.92 M/ul	0.11 M/ul
HCT	19.2%	1.4%
MCV	208.7 fl	127.3 fl
MCH	44.6 pg	436.4 pg
MCHC	21.4 g/dl	342.9 g/dl
Total WBC count	65,190/cumm	57,890/cumm

Measurement units: grams per deciliter (g/dl), millions per cubic milimeter (M/ul), femtoliters (fl), picograms (pg), per cubic milimeter (/cumm).

**Table 5:** Blood parameters for hypoxic condition.

Blood parameters	Control	Test
Haemoglobin	4.1 g/dl	4.8 g/dl
RBC count	0.92 M/ul	0.09 M/ul
HCT	19.2%	1.0%
MCV	208.7 fl	129.3 fl
MCH	44.6 pg	400.4 pg
MCHC	21.4 g/dl	300.9 g/dl
Total WBC count	65,190/cumm	54,890/cumm

## Conclusion

This study focuses on to give an ideal and easy method to determine the behavioural changes in goldfish with respect to change in real-time temperature and DO. To perform this study, the water temperature was raised from 28 to 42 °C, with a rising of 2 °C per 8 h interval. Mainly three behavioural changes were noticed i.e., resting at bottom, gasping and erratic swimming behaviour. Total of 146 instances were recorded where fish shown behaviour change. Each instance was marked by real time water temperature and dissolved oxygen data. The dependent behavioural data classified against the independent real-time temperature and DO data. For this purpose, four classifiers were used i.e., decision tree, Naïve Bayes classifier, KNN, and Linear discriminant analysis. K (= 10) fold validation method and confusion matrix were used to compare the performance between all four classifiers. The cross-validation error for the decision tree was lowest (13.78%), while KNN had the highest error (30.14%). Here, the decision tree was proved to be efficient compared to other classifiers, so it was used to classify the behavioural change data. The decision tree was of six terminal nodes that can predict the behavioural changes with respect to changes in temperature and DO. To validate the external behavioural changes of fish with their physiological behavioural changes, tests were done on blood parameters and compared between control and test fish to show the changes in fish blood.

This is a pilot study and in future it can be expanded to the field level in the different ornamental fish farms; other fresh water, marine and coastal fish farms like the shrimp farm, cages, and pens. For further studies on machine learning and deep learning, different methods can be done like fish counting and tracking, estimation of swimming speed, abnormal behaviour detection, speed change detection, etc. The studies can be done in a real-time identification automated system for fish disease diagnosis. One can upgrade the sensors for more water quality parameters like ammonia, nitrite, and nitrate and collection of more data that can be used for big data and analytics or to develop some AI algorithms for process optimization. The fish detection and tracking methods can be used while doing sampling in freshwater or marine environments.

## Data Availability

The utilized data in this study is available upon reasonable request from the corresponding author.
